# KneeTex: an ontology–driven system for information extraction from MRI reports

**DOI:** 10.1186/s13326-015-0033-1

**Published:** 2015-09-07

**Authors:** Irena Spasić, Bo Zhao, Christopher B. Jones, Kate Button

**Affiliations:** School of Computer Science & Informatics, Cardiff University, Cardiff, CF24 3AA UK; School of Healthcare Sciences, Cardiff University, Cardiff, CF14 4XN UK

## Abstract

**Background:**

In the realm of knee pathology, magnetic resonance imaging (MRI) has the advantage of visualising all structures within the knee joint, which makes it a valuable tool for increasing diagnostic accuracy and planning surgical treatments. Therefore, clinical narratives found in MRI reports convey valuable diagnostic information. A range of studies have proven the feasibility of natural language processing for information extraction from clinical narratives. However, no study focused specifically on MRI reports in relation to knee pathology, possibly due to the complexity of knee anatomy and a wide range of conditions that may be associated with different anatomical entities. In this paper we describe KneeTex, an information extraction system that operates in this domain.

**Methods:**

As an ontology–driven information extraction system, KneeTex makes active use of an ontology to strongly guide and constrain text analysis. We used automatic term recognition to facilitate the development of a domain–specific ontology with sufficient detail and coverage for text mining applications. In combination with the ontology, high regularity of the sublanguage used in knee MRI reports allowed us to model its processing by a set of sophisticated lexico–semantic rules with minimal syntactic analysis. The main processing steps involve named entity recognition combined with coordination, enumeration, ambiguity and co–reference resolution, followed by text segmentation. Ontology–based semantic typing is then used to drive the template filling process.

**Results:**

We adopted an existing ontology, TRAK (Taxonomy for RehAbilitation of Knee conditions), for use within KneeTex. The original TRAK ontology expanded from 1,292 concepts, 1,720 synonyms and 518 relationship instances to 1,621 concepts, 2,550 synonyms and 560 relationship instances. This provided KneeTex with a very fine–grained lexico–semantic knowledge base, which is highly attuned to the given sublanguage. Information extraction results were evaluated on a test set of 100 MRI reports. A gold standard consisted of 1,259 filled template records with the following slots: finding, finding qualifier, negation, certainty, anatomy and anatomy qualifier. KneeTex extracted information with precision of 98.00 %, recall of 97.63 % and F–measure of 97.81 %, the values of which are in line with human–like performance.

**Conclusions:**

KneeTex is an open–source, stand–alone application for information extraction from narrative reports that describe an MRI scan of the knee. Given an MRI report as input, the system outputs the corresponding clinical findings in the form of JavaScript Object Notation objects. The extracted information is mapped onto TRAK, an ontology that formally models knowledge relevant for the rehabilitation of knee conditions. As a result, formally structured and coded information allows for complex searches to be conducted efficiently over the original MRI reports, thereby effectively supporting epidemiologic studies of knee conditions.

## Background

Magnetic resonance imaging (MRI) is a technique used to visualise internal body structure by recording radio waves emitted by the tissues in the presence of a strong magnetic field. MRI better differentiates between soft tissues than does X-ray imaging, which uses high frequency electromagnetic waves that pass through soft parts of the human body to create a radiograph, an image resulting from the different absorption rates of different tissues. MRI can also produce three dimensional images. When it comes to diagnosing knee pathology, MRI has the advantage of visualising all structures within the knee joint, i.e. both soft tissue and bone. When used in conjunction with medical history and physical examination, this makes MRI a valuable tool for increasing diagnostic accuracy and planning surgical treatments [1–5]. For example, meniscal tears are a relatively common knee injury, having a prevalence of 22.4 % among all soft tissues injuries seen in a trauma department [6]. The accuracy of diagnosing meniscal tears using individual physical tests is reported to be 74 %, but increases to 96 % when MRI is used [5]. When MRI results are combined with clinical assessments (namely, locking, giving way and McMurray’s test), then their diagnostic performance increases respectively as follows: accuracy – 88.3 %, 89.9 % and 89.4 %, sensitivity – 95.7 %, 97.4 % and 97.4 %, specificity – 74.2 %, 75.8 % and 74.2 %, positive predictive value – 87.5 %, 88.4 % and 87.7 %, and negative predictive value – 90.2 %, 94.0 % and 93.9 % [4]. More recently, the importance of MRI in diagnosis and treatment planning for cases of symptomatic early knee osteoarthritis has been highlighted. If an X–ray image of the knee is found to be normal, but clinical examination produces specific findings, then MRI scan can be performed to establish more accurate diagnosis. It can be used to identify an appropriate surgical or nonsurgical treatment target and decrease the need for costly and invasive diagnostic arthroscopy [1, 7].

In clinical practice, radiology images (e.g. produced by X–ray or MRI) are usually accompanied by imaging reports (or radiology reports), which serve the purpose of conveying a specialist interpretation of images and relate it to the patient’s signs and symptoms in order to suggest diagnosis [8]. This information is then used by clinicians to support decision making on appropriate treatment.

In terms of research, MRI evidence is often used to support epidemiologic studies of knee pathology [9, 10]. In particular, MRI findings are indispensible features of longitudinal studies of knee osteoarthritis [11, 12], where lesions detected by MRI were found to precede onset of clinical symptoms. However, many of published research findings are probably false due to sampling bias and low statistical power [13]. Small sample size is often the cause underlying these two concerns although the relationship is not simple or proportional [14]. Unfortunately, sample size is typically subject to funding and personnel constraints. Given the complexity and cost of manual interpretation of MRI evidence, it is, therefore, not surprising that the size of such epidemiologic studies has been limited to hundreds (e.g. 514 [9], 710 [10]) or even dozens of cases (e.g. 20 [11], 36 [12]). If interpretation of evidence described in MRI reports could be automated, then it would overcome the size limitation in retrospective cohort studies posed by the need to manually sort through the evidence.

We recently provided a critical overview of the current state of the art for natural language processing (NLP) related to cancer [15], where clinical narratives such as those found in pathology and radiology reports convey valuable diagnostic information that is predictive of the prognosis and biological behaviour of a disease process [16]. The review highlighted the fact that a range of studies have proven the feasibility of NLP for extracting structured information from free text reports (e.g. [17–21]). For simpler information extraction tasks, human–like performance of automated systems can be expected. For example, when evaluated for the extraction of American College of Radiology utilisation review codes from radiology reports, M+, a system for medical text analysis, achieved recall, precision and specificity of 87, 85 and 98 % respectively [22]. These results were comparable to average recall, precision and specificity recorded by physicians, namely 88, 86 and 98 %. Comparably good results were achieved for more complex tasks such as translating radiology reports into a large database [18], where the Medical Language Extraction and Encoding (MedLEE) system achieved recall of 81 % and specificity of 99 % with a total of 24 clinical conditions (diseases, abnormalities and clinical states) being the subject of the study. Again these results were comparable to average recall (85 %–87 %) and specificity (98 %) achieved by expert human coders.

Typical processing steps taken in such NLP systems include text segmentation into words, sentences, paragraphs and/or sections, part–of–speech tagging, parsing, named entity recognition (NER), normalisation and negation annotation [17, 23, 24]. Recognition of named entities, i.e. phrases that are used to differentiate between entities of the same semantic type (e.g. *Osgood-Schlatter disease* is a name used to refer to a specific *disease*), followed by normalising the representation of their meaning (e.g. *Osgood-Schlatter disease* is also known as *apophysitis of the tibial tubercle* or *OSD*), is the crucial step towards semantic interpretation of clinical narratives. In order to disambiguate named entities and assert relationships between them (e.g. relate disease/disorder, sign/symptom or procedure to an anatomical site), domain–specific knowledge needs to be available in a machine–readable form. For example, the domain knowledge is specified in MedLEE using a table created manually based on domain expertise [17]. Similarly, Medical Text Analysis System (MedTAS) utilises external knowledge resources such as terminologies and ontologies [25]. Alternatively, M+ uses Bayesian Networks to represent semantic types and relations within a specific medical domain such as that of chest radiology reports [22]. Ideally, when a suitable ontology is available it can be used to add an explicit semantic layer over text data by linking domain–specific terms, i.e. textual representation of concepts, to their descriptions in the ontology [26]. This allows text to be mined for interpretable information about domain–specific concepts and their relationships.

In our previous work, we developed TRAK (Taxonomy for RehAbilitation of Knee conditions), an ontology that formally models knowledge relevant for the rehabilitation of knee conditions [27]. This knowledge resource allowed us to implement an NLP system able to interpret knee–related clinical findings from MRI reports. In this paper, we describe KneeTex, an open–source, stand–alone application developed to address the task of information extraction from narrative reports that describe an MRI scan of the knee. KneeTex is an ontology–driven, rule–based system. It takes an MRI report as an input and outputs the corresponding clinical findings in the form of JavaScript Object Notation (JSON) objects, a lightweight data–interchange format [28]. KneeTex not only extracts, but also codes the extracted information by mapping it onto the TRAK ontology. The resulting formally structured and coded information allows complex searches to be conducted efficiently over the original MRI reports, thereby effectively supporting epidemiologic studies of knee conditions.

## Methods

### System specification

**Fig. 1:**
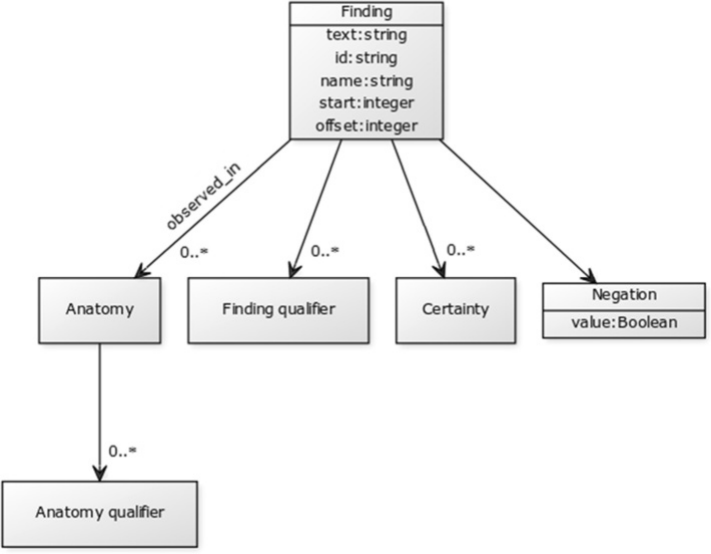
Information extraction template represented by UML diagram. Each slot has got the following properties: extracted text, concept identifier, preferred concept name, start position of extracted text and its length

Information extraction (IE) is the task of automatically selecting specific facts about pre–specified types of entities and relationships from free–text documents. In other words, the goal of IE is to convert free text into a structured form by filling a template (a data structure with predefined slots) with the relevant information extracted (slot fillers) [29]. Figure 1 provides a graphical representation of a template specific to our system, whose structure is illustrated using Unified Modelling Language (UML) [30]. The template specifies the types of entities and relationships we aim to extract in this particular study.

The goal of our system is to extract information about clinical observations made from MRI scans. Information extracted about individual observation is structured into two major parts: finding and anatomy. Finding represents a clinical manifestation (e.g. injury, disease, etc.) observed by a radiologist. In other words, it corresponds to what is observed. Information related to anatomy refers to a specific part of human anatomy affected by the finding. In other words, it corresponds to where the finding is observed. Both finding and anatomy may have qualifiers, which provide more specific information extracted about them. In addition to general qualifiers, each finding is associated with information about its certainty (as judged by the radiologist) and negation (which specifies whether the finding is positive or negative). Tables 1 and 2 provide examples of a filled template based on information extracted automatically from the given sentences. The filled template examples are represented using JSON.

**Table 1:**
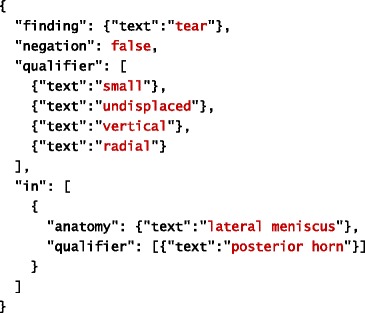
An example of a filled template. Original text source: *“There is a small undisplaced vertical radial tear of the posterior horn of the lateral meniscus.”*

**Table 2:**
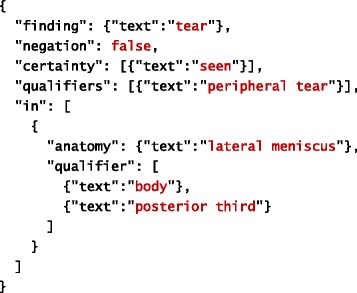
An example of a filled template. Original text source: *“A peripheral tear involving the body of the lateral meniscus extending into the posterior third is seen.”*

### Data

Between January 2001 and May 2012, a total of 6,382 individuals with an acute knee injury attended the Acute Knee Screening Service at the Emergency Unit of the Cardiff and Vale University Health Board (C&V UHB). A subset of 1,657 individuals fulfilled locally agreed clinical criteria for an MRI scan. Both the clinical assessment and MRI findings for these individuals were stored in a clinical database on a secure server within the C&V UHB. This database was originally developed for the purposes of service evaluation and auditing practice. Out of 1,657 referred individuals, a total of 1,468 MRI scan visits were identified retrospectively from the database records. Following an MRI scan, the imaging results were summarised by a radiologist (from a team of five) in a diagnostic narrative report that conveys a specialist interpretation of the MRI scan and relates it to the patient’s signs and symptoms. These MRI reports formed the dataset used in this study.

All reports were anonymised by removing all identifiable information related to either patient or radiologist together with the attendance date and the links to the patient’s assessment and treatment details. The anonymised reports were transferred to an encrypted memory stick that was password protected and locked in a filing cabinet in a lockable room. Ethical approval for this study was obtained from the South East Wales Research Ethics Committee (10/MRE09/29).

As part of their radiology reporting initiative whose aim is to improve reporting practices by creating a library of clear and consistent report templates, the Radiological Society of North America provides a template for knee MRI reports [31]. However, the reports in our dataset did not follow any such predefined structure. The structure varied across the reports, but they generally tended to organise information under the following headings: mri of the left/right knee, indication, history, findings and conclusion. Within the reports, which were distributed in a plain text format, these sections were indicated with upper case (see Table 3 for an example).

**Table 3:**
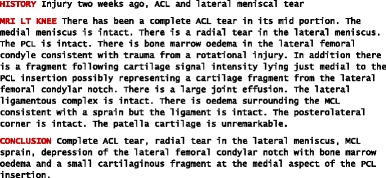
Knee MRI report. A sample from the training dataset

The size of the overall dataset was 1,002 KB with a total of 13,991 sentences, 178,931 tokens, 3,277 distinct tokens and 2,681 distinct stems. On average, the size of an individual MRI report was 0.68 KB (±0.40 KB) with a total of 9.53 (±5.13) sentences and 110.81 (±64.60) tokens.^1^ We separated the data into training and testing sets. A test set was created by randomly selecting a subset of 100 MRI reports from the overall dataset. These reports were then removed from consideration so that the performance of the system could later be evaluated on unseen data. The remaining 1,368 reports formed a training set, which was used to inform system development.

**Fig. 2:**
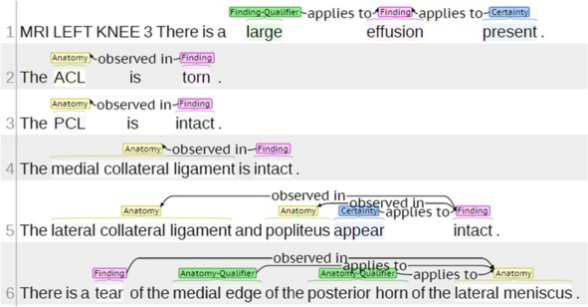
A manually annotated MRI report. A screenshot of the BRAT interface

For training and testing purposes, the data were manually annotated with labels that correspond to slots and their relationships from the IE template (see Fig. 1). The annotation was performed using BRAT, a web–based tool for text annotation [32]. The process involved annotating text spans with slot names (e.g. *finding* or *anatomy*) as well as annotating dependencies between them (e.g. *observed*_*in*). Figure 2 provides a visualisation of an annotated example. A total of 100 training documents and 100 testing documents were annotated independently by two annotators.

### Ontology

We previously developed TRAK as an ontology that formally models knowledge relevant for the rehabilitation of knee conditions [27]. This information includes classification of knee conditions, detailed knowledge about knee anatomy and an array of healthcare activities that can be used to diagnose and treat knee conditions. Therefore, TRAK provides a framework that can be used to collect coded data in order to support epidemiologic studies much in the way Read Codes, a coded thesaurus of clinical terms [33], are used to record observational data in the Clinical Practice Research Datalink (CPRD) – formerly known as the General Practice Research Database (GPRD) [34]. TRAK follows design principles recommended by the Open Biomedical Ontologies (OBO) Foundry and is implemented in OBO [35], a format widely used by this community. Its public release can be accessed through BioPortal [36], a web portal that provides a uniform mechanism to access biomedical ontologies, where it can be browsed, searched and visualised.

TRAK was initially developed with a specific task in mind – to formally define standard care for the rehabilitation of knee conditions. At the same time, it was designed to be extensible in order to support other tasks in the domain. For example, the knowledge about knee anatomy, which is cross–referenced to a total of 205 concepts in the Foundational Model of Anatomy (FMA) [37], is directly applicable to interpretation of reports describing knee MRI scans. However, in order to fully support semantic interpretation of this type of clinical narratives, the TRAK ontology needed to be expanded with other types of MRI–specific concepts.

### Ontology expansion

In order to support semantic interpretation of the terminological content found in knee MRI reports, we needed to ensure that all relevant concepts are modelled appropriately in the TRAK ontology. The main aspect of this task was the expansion of a specific domain modelled by the ontology, for example, MRI–specific observations such as *hyaline cartilage abnormality, bone bruise, cyclops lesion*, etc. In order to support NLP applications of the ontology, its vocabulary also needed to be expanded to include term variants commonly used in MRI reports. Some term variants are confined to a specific clinical sublanguage [18] and as such are typically underrepresented in standardised medical dictionaries such as those included in the Unified Medical Language System (UMLS) [38]. For example, *collateral ligament* was found to have no other synonyms in the UMLS. Yet, *collateral ligaments* are colloquially referred to as *collaterals* in clinical narratives. Thus, out of 37 references to *collateral ligaments* in the training dataset, six (i.e. 16 %) accounted for this informal variant of the term.

We devised four strategies for systematic expansion of the coverage of the TRAK ontology. Three of these strategies were data–driven. This was to ensure that the ontology is appropriate for the intended NLP applications on such data. Each data–driven strategy utilised a different approach to extracting the relevant terminology from the data either manually or automatically. The fourth strategy was based on integration of known concepts from other relevant knowledge sources. The two main aims of this strategy were: (1) to avoid overfitting the ontology based on limited data used in the data–driven strategies, and (2) to provide an initial taxonomic structure to incorporate new concepts.

**Fig. 3:**
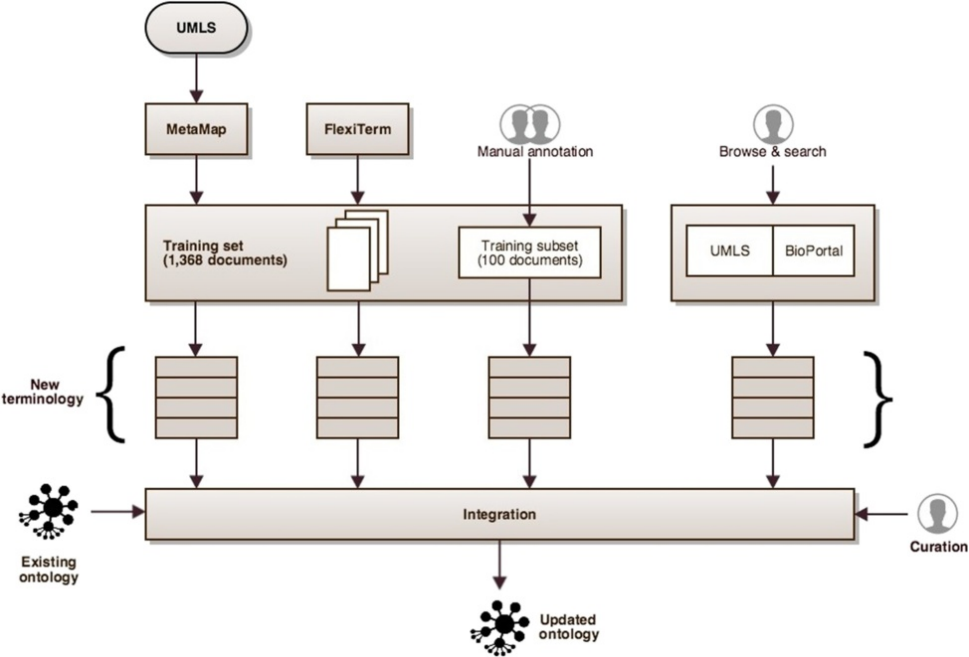
The strategies for rapid ontology expansion. Newly identified terminology is integrated into the existing ontology

The four strategies were applied independently and their results were subsequently integrated (see Fig. 3). As a result, the original TRAK ontology expanded from 1,292 concepts, 1,720 synonyms and 518 relationship instances to 1,621 concepts, 2,550 synonyms and 560 relationship instances. The following sections outline each strategy in more detail.

### Strategy 1: dictionary-based term recognition

**Fig. 4:**
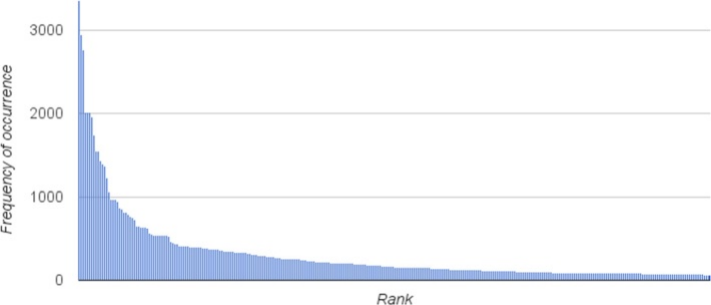
Distribution of UMLS concept mentions. MetaMap was used to automatically identify concept mentions in the training set

The vast majority of existing TRAK concepts (more precisely, 875 out of 1,292) were originally cross–referenced to the UMLS, a terminological resource that integrates over 150 biomedical vocabularies [38], in an attempt to standardise TRAK terminology and facilitate its integration with other terminological sources. During the initial development of TRAK, the UMLS was searched collaboratively by a physiotherapist (who was both practitioner and researcher) and an informatician to obtain concept identifiers, synonyms and definitions, where such information was available. Given the availability of MRI reports, we were now able to automate the process of finding other relevant concepts in the UMLS. For this purpose, we used MetaMap, a software tool for recognising UMLS concepts in biomedical text [39]. We applied MetaMap against a training corpus of 1,368 MRI reports to recognise UMLS concepts and obtain their unique concept identifier and a preferred name in the UMLS. Given that the majority of TRAK concepts (approximately 68 %) were already cross–referenced to the UMLS, we used these identifiers to automatically remove known UMLS concepts from unnecessary consideration. The remaining MetaMap output formed a list of 1,121UMLS concepts to be considered for inclusion in TRAK. To facilitate the manual curation process, the list was ordered by the frequency of occurrence of each concept within the training dataset. The frequency graph shown in Fig. 4 depicts a power law distribution [40] of UMLS concept mentions. Using the Pareto principle (or 80:20 rule) as a guideline [41], we focused on approximately 20 % of most frequently mentioned concepts by considering only those that occurred at least 100 times in the training dataset. A total of 215 frequently mentioned UMLS concepts were manually curated and considered for inclusion in TRAK. Some examples of highest ranked relevant concepts include *intact, rupture, laceration*, etc.

### Strategy 2: automatic term recognition

Using the UMLS to identify relevant concepts in text data has the advantage of providing not only their definitions and synonyms, but also their classification and a potential structure into which to embed them within the TRAK ontology. However, a previous lexical study conducted on a large corpus of various types of medical records (discharge summaries, radiology reports, progress notes, emergency room reports and letters) revealed that clinical narratives are characterised by a high degree of misspellings, abbreviations and non–standardised terminology [42]. The given study found that over 20 % of the words used were unrecognisable, i.e. were not recognisable medical words, common words or names, and could not be algorithmically or contextually converted to such words. However, almost 78 % of unrecognisable words were judged to be probably correctly spelled medical terms. These findings illustrate the challenges clinical narratives pose to dictionary–based term recognition methods such as that implemented by MetaMap.

In order to extract additional terms from the training dataset that were not found in the UMLS, we complemented the use of MetaMap with FlexiTerm, our own data–driven method for automatic term recognition from a domain–specific corpus [43]. For the original publication, we thoroughly evaluated FlexiTerm on five biomedical corpora including a subset of 100 MRI reports from the dataset used in this study. The highest values for precision (94.56 %), recall (71.31 %) and F-measure (81.31 %) were achieved on this particular corpus.

FlexiTerm performs recognition of multi–word terms in two steps: linguistic filtering is used to select term candidates followed by calculation of termhood, a corpus–based measure that combines strength of collocation with frequency of occurrence. Termhood values are used as evidence to select higher–ranked candidates as terms over the lower–ranked ones. In order to improve statistical distribution of termhood values, which may be affected by term variation phenomena, FlexiTerm uses a range of methods to neutralise the main causes of term variation and thereby aggregate termhood values that would otherwise be dispersed across different variants of the same term. Firstly, FlexiTerm manages syntactic variation by processing term candidates using a bag–of–words approach. Further, orthographic and morphological variations are neutralised by stemming in combination with lexical and phonetic similarity measures. Consequently, FlexiTerm not only extracts terms from text, but it also groups term variants such as *infrapatellar fat pad, infra-patella fat pad* and *infra-patellar fat pad* together. This allows for identification of new concepts (e.g. *posterior horn* ranked seventh by FlexiTerm was added as a new concept in TRAK), but also identification of previously unknown names of existing concepts, which are easily mapped to a concept via its known names. For example, *lateral femoral condyle* was identified as a new synonym for a concept with identifier TRAK:0001037 previously known only as *lateral condyle of femur*.

**Fig. 5:**
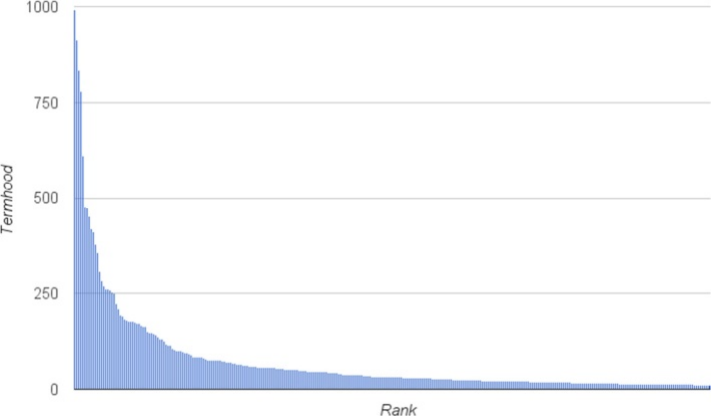
Distribution of termhood. FlexiTerm was used to automatically identify multi–word terms in the training set

We ran FlexiTerm over the whole training dataset of 1,368 MRI reports and extracted 1,076 term candidates with a total of 1,422 term variants. To facilitate the manual curation process, the list of automatically extracted terms was ordered by their termhood calculated by FlexiTerm. Similarly to the frequency graph shown in Fig. 4, the termhood graph shown in Fig. 5 also depicts a power law distribution. Therefore, relying on the Pareto principle again, we focused on approximately 20 % of highest ranked terms by considering only those with termhood over 20. A total of 222 automatically extracted terms were manually curated and considered for inclusion in TRAK.

### Strategy 3: manual data annotation

As part of developing and testing our information extraction system, we manually annotated the test set and a portion of the training set. The annotated test set was later used to create a gold standard to evaluate the system, whereas an annotated subset of 100 training documents was used not only to test the system during its development, but also to inform the expansion of the ontology with terms manually annotated in text. This strategy offers a potential to identify additional concepts and their names, particularly those that are non–standardised and occur less frequently in the training dataset. Recall that MetaMap identifies concepts based solely on the content of standardised medical dictionaries included in the UMLS. On the other hand, FlexiTerm may identify some non–standardised terminology, but in doing so it relies on the frequency of term occurrence. Moreover, FlexiTerm only extracts multi–word terms, thus ignoring concepts designated by a single word (e.g. *fissure, ganglion*, *etc*.). In addition to enabling us to identify relevant concepts overlooked by the previous two strategies, the annotation exercise allowed us to explore in detail how the terms were used in context, which helped disambiguate their meaning based on which they were embedded into the existing ontology structure.

The annotation categories relevant for the manual identification of terms include *finding* (an observation such as the presence of a disease or an injury), *finding qualifier* (further specification of the finding), *certainty* (observational evidence used to assert the finding), *anatomy* (anatomical entity such as tissue or an organ to which the finding applies) and *anatomy qualifier* (further specification of the affected anatomical entity). For example, in the following sentence:

*There isdefinite complex tearingof theposterior hornandbodyof themedial meniscus.*

*tearing* represents the finding, *definite* its certainty, *complex* its qualifier, *medial meniscus* the anatomical entity affected, whereas *posterior horn* and *body* are anatomy qualifiers that provide more specific location for the given finding.

A total of 484 unique phrases (not necessarily terms) with 2,071occurrences were annotated as instances of *finding*, 113 unique phrases with 284 occurrences as instances of *finding qualifier*, 68 unique phrases with 202 occurrences as instances of *certainty*, 208 unique phrases with 1,232 occurrences as instances of *anatomy*, and finally 178 unique phrases with 469 occurrences as instances of *anatomy qualifier*. The fact that these phrases were pre–classified into four broad categories allowed us to focus on particular branches of the TRAK hierarchy one at a time. In addition, some categories (e.g. *anatomy* and *anatomy qualifier*) were already extensively covered by the TRAK ontology. Therefore, the removal of 137 known terms referring to 60 TRAK concepts from unnecessary consideration greatly facilitated the manual curation and allowed us to consider all remaining phrases for potential inclusion in TRAK.

### Strategy 4: manual dictionary search

So far, all three strategies for identification of new ontology concepts relied on the training dataset from which candidates were selected using a combination of automatic and manual methods. These data–driven approaches runs a risk of overfitting the ontology based on the available data, which may result in incomplete coverage of the domain simply because some concepts (possibly the ones less frequently encountered in practice) were not mentioned in the available sample of MRI reports. In order to systematically cover the domain by including potentially relevant concepts that are not seen in the training dataset, we consulted two authoritative knowledge sources relevant for semantic interpretation of MRI reports.

**Fig. 6:**
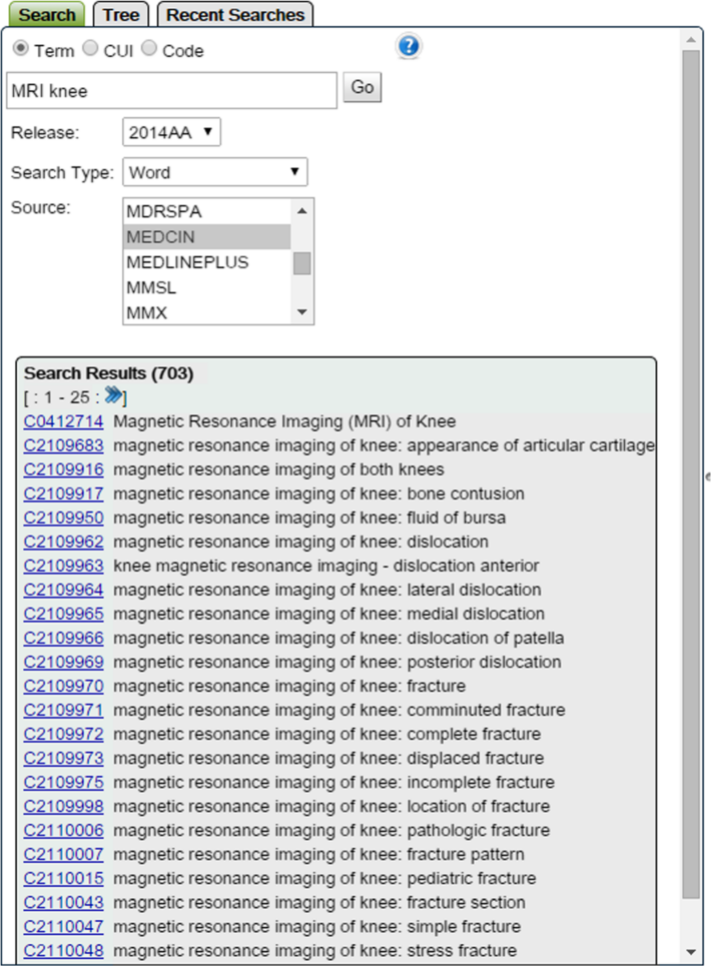
MEDCIN terminology related to MRI of knee. UMLS terminology services were used to access relevant terminology

The first source, MEDCIN, was identified through the UMLS terminology services [44]. MEDCIN is a medical terminology created and maintained by Medicomp Systems, Inc. as part of their system for management of clinical information [45, 46]. MEDCIN contains more than 250,000 concepts encompassing symptoms, history, physical examination, tests, diagnoses and therapies structured into multiple clinical hierarchies. One such hierarchy with the root element named *magnetic resonance imaging of knee* represents a detailed taxonomy of findings that can be identified from knee MRI scans. We extracted this particular taxonomy from the UMLS by using *MRI knee* as a search term restricted to MEDCIN as the source vocabulary (Fig. 6 illustrates the search results).

All 703 concepts extracted from MEDCIN were named using phrases that represent detailed descriptions rather than traditional terms. After removing the common header, *magnetic resonance imaging of knee*, from these phrases, we decomposed them into four categories: *finding, finding qualifier, anatomy* and *anatomy qualifier*. For example, in the following example taken from MEDCIN:

*magnetic resonance imaging of knee:acute osteochondral injuryof posterior aspect of lateral femoral condyle*

*osteochondral injury* represents the finding, *acute* its qualifier, *lateral femoral condyle* the anatomical entity affected, whereas *posterior aspect* is its qualifier, which provides more specific location for the given finding. Most anatomical concepts were already covered in TRAK, so the manual curation process focused mainly on concepts related to findings and their qualifiers. The resulting list consisted of 76 concepts, which were then manually curated.

**Fig. 7:**
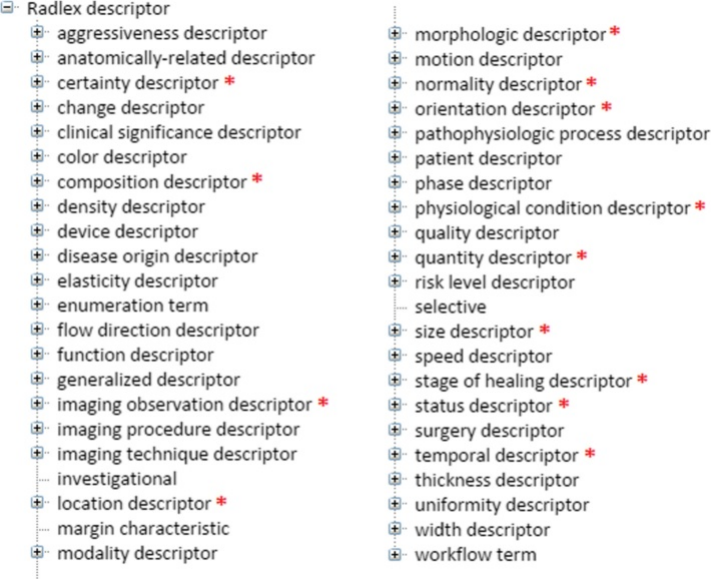
RadLex terminology related to descriptions of radiology findings. BioPortal was used to access relevant terminology

The second source, RadLex, was identified through BioPortal, the most comprehensive repository of biomedical ontologies [47]. MRI is a technique used in radiology, a medical specialty whose concepts are formally described in the Radiology Lexicon (RadLex) – a controlled terminology designed as a single unified source of terms for radiology practice, education and research in an attempt to fill in the gaps in other medical terminology systems [48]. RadLex is currently not distributed as a part of the UMLS. A study conducted on a corpus of 800 radiology reports that represented a mixture of imaging modalities including MRI revealed that out of 11,962 mentioned terms found in RadLex, 3,310 terms (i.e. almost 28 %) could not be found in the UMLS [49]. These facts imply that much of the RadLex terminology would not be identified by MetaMap, used previously to identify UMLS terms. We systematically explored RadLex using its distribution via BioPortal [36]. In particular, we focused on the *RadLex descriptor* branch of the RadLex hierarchy (see Fig. 7), whose leaf node children are mainly adjectives (rather than noun phrases, which is customary for terms) that can be used to describe radiology findings by specifying their qualifiers (e.g. *lobulated* would be a qualifier of a *cyst*). We considered a total of 41 subclasses out of which 13 were relevant for MRI reports (these classes are indicated with an asterisk in Fig. 7). These subclasses were used not only as the source of potential terms for TRAK, but also to provide a structure for incorporating such terms into TRAK. As the end result, a total of 167 terms were cross-referenced to RadLex. The *RadLex descriptor* class has been renamed to *finding descriptor* and embedded into TRAK as a subclass of *quality*.

### Information extraction

In addition to extracted text (see Tables 1 and 2), the JSON schema we created to formally model the IE template shown in Fig. 1 prescribes the following properties for each slot:
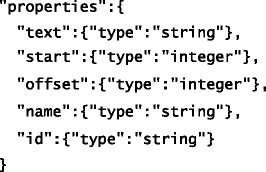


As before, text refers to the actual content extracted from text, whereas start and offset refer to its location in the document. The extracted information is further mapped onto the corresponding concept in the TRAK ontology, whose identifier is stored within the id property. The concept’s preferred name is retrieved from the ontology and used to complete the name property in order to facilitate interpretation of extracted information. Given this more precise description of the IE task, Tables 4 and 5 provide examples of a filled template based on information extracted automatically from the given sentences. As before, the filled template examples are represented using JSON. For simplicity reasons, we omitted start and offset properties from these illustrations.

**Table 4:**
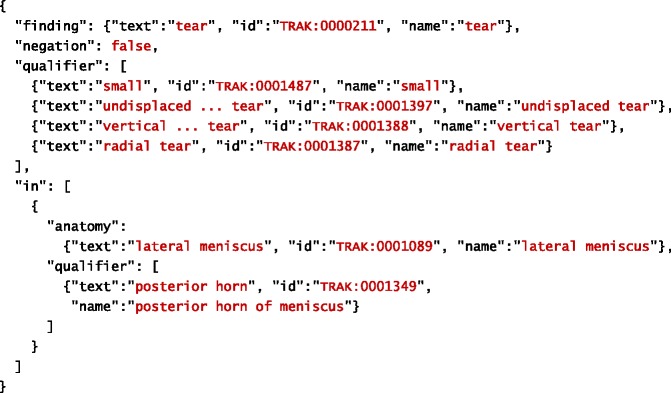
An example of a filled template. Original text source: *“There is a small undisplaced vertical radial tear of the posterior horn of the lateral meniscus.”*

**Table 5:**
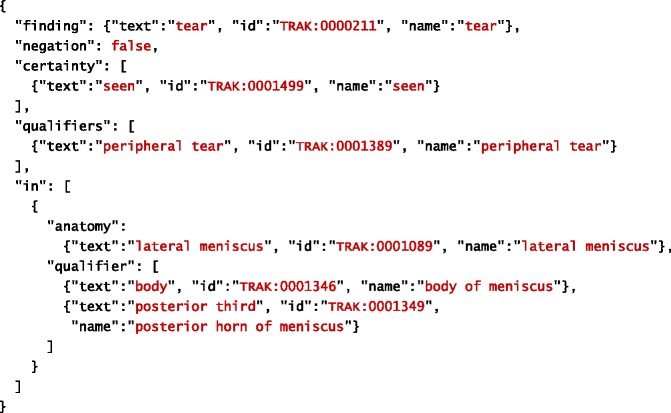
An example of a filled template. Original text source: *“A peripheral tear involving the body of the lateral meniscus extending into the posterior third is seen.”*

Once coded, the extracted information can be searched systematically. For instance, note that in the given examples equivalent phrases, *posterior horn* and *posterior third*, were mapped to the same concept, which allows for the extracted information to be searched by the underlying meaning and not merely its surface realisation in text. Note that KneeTex is an IE system and as such does not include an interface to search through the extracted information. However, the JSON format of extracted information allows for it to be stored directly into a document–oriented database such as MongoDB [50], from which it can be easily queried.

**Fig. 8:**
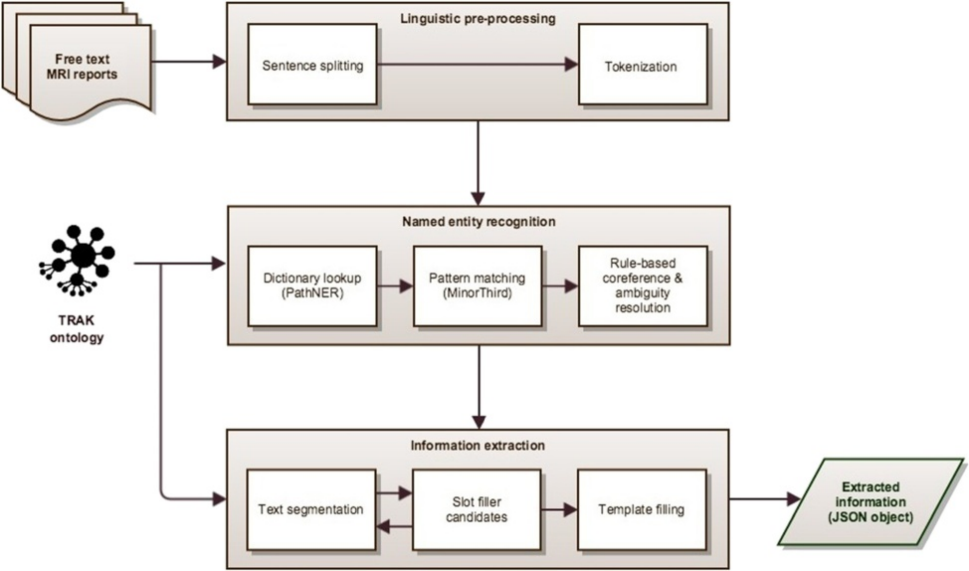
The system architecture diagram. TRAK ontology and all intermediate results are saved in a relational database to enable their integrative querying

Figure 8 depicts the overall system architecture with specific modules described in more detail in the following sections.

### Linguistic pre-processing

Previous lexical analysis of a large corpus of various types of clinical narratives (discharge summaries, radiology reports, progress notes, emergency room reports and letters) revealed that they are characterised by a high degree of misspellings, abbreviations and idioms [42]. However, the analysis of our training corpus revealed a total of 1,138 typographical errors averaging at 0.83 errors per document. The low percentage of typographical errors was not expected to significantly hinder subsequent processing. Therefore, we supported only traditional elements of linguistic pre–processing (i.e. tokenisation and sentence splitting) in this module and dealt with typographical errors and spelling mistakes by choosing a method for named entity recognition that is robust against such variations.

### Dictionary lookup

Having sufficiently expanded the original TRAK ontology, its vocabulary can now be used to drive named entity recognition, whose aim is to automatically identify and classify words and phrases into predefined categories such as diseases, symptoms, anatomical entities, etc. In effect, NER is used here to identify candidates for slot fillers and as such represents the main vehicle of IE. The performance of dictionary–based NER approaches varies across different dictionaries and tools. A recent evaluation of three such state–of–the–art tools on a set of eight biomedical ontologies showed that their performance in terms of F–measure varied from 14 % to 83 % [51]. ConceptMapper (a component of the Apache UIMA Sandbox [52]) generally provided the best performance. Beside performance, we considered the ease of use. While converting an OBO ontology to ConceptMapper’s dictionary format is straightforward, one must adopt the UIMA framework in order to use this particular component. For flexibility reasons, we opted to use PathNER [53] as an alternative to ConceptMapper.

PathNER (Pathway Named Entity Recognition) is a freely available tool originally developed for systematic identification of pathway mentions in the literature. On a pathway–specific gold–standard corpus, PathNER achieved F–measure of 84 % [51]. It implements soft dictionary matching by utilising the SoftTFIDF method [54], a combination of the term frequency–inverse document frequency (TF–IDF) [55] and the Jaro–Winkler distance [56]. This makes the dictionary lookup robust with respect to the problem of term variation commonly seen in biomedical text, which often causes dictionary lookup based on exact string matching to fail [57]. Typical term variations include morphological variation, where the transformation of the content words involves inflection (e.g. *lateral meniscus* vs. *lateral menisci*) or derivation (e.g. *meniscus tear* vs. *meniscal tear*), and syntactic variation, where the content words are preserved in their original form (e.g. *apex of patella* vs. *patella apex*) [43].

In order to use PathNER to identify TRAK terms in text, we extracted the vocabulary from the ontology and converted it into PathNER’s internal dictionary format. In effect, PathNER is used here to identify TRAK terms in text as candidates for slot fillers and as such represents the basis for template filling. We identified a few potential issues in the context of the given template. These relate in particular to the fact that the template requires two distinct main types of named entities: anatomical entities (e.g. *organ*, *tissue*, etc.) and findings (e.g. *injury*, *disease*, etc.). In order to systematically classify knee conditions, TRAK incorporates a knee–relevant portion of the Orchard Sports Injury Classification System (OSICS) Version 10 [58]. OSICS–10 is a classification system in which all classes encompass two types of information: (1) type of condition (injury or disease) and (2) anatomical entity affected by the condition. Our own approach to formal modelling of knee conditions was to separate these two aspects and represent them by two distinct semantic types that correspond to finding and anatomy. For example, TRAK incorporates the following three terms: *ACL rupture*, *ACL* and *rupture* (see Table 6 for details).

**Table 6:**
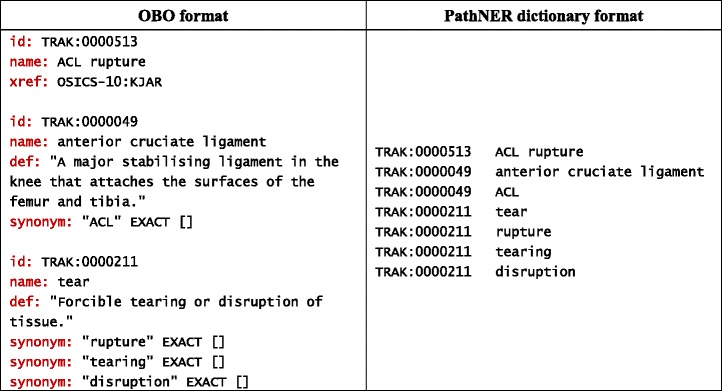
An excerpt from the TRAK ontology. Exporting ontology vocabulary into PathNER’s dictionary format

Obviously, the term *ACL rupture*, originally imported from OSICS–10, encompasses the other two terms. While the nature of taxonomic classification taken in OSICS–10 is useful for a range of applications in epidemiologic research [59], it may pose problems for NER en route to template filling. Namely, PathNER looks for the longest possible match. This means that, given the three dictionary entries, the longest match in the following sentence:

*HISTORY Twisting injury, ACL rupture and medial meniscal tear.*

would result in the following annotation:

*HISTORY Twisting injury, <term id = “0000513” > ACL rupture</term > and medial meniscal tear.*

Alternatively, two separate annotations of *ACL* and *rupture* as follows:

*HISTORY Twisting injury, <term id = “0000049” > ACL</term > <term id = “0000211” > rupture</term > and medial meniscal tear.*

would greatly simplify the process of template filling, since the two recognised named entities can be mapped directly to the corresponding slots in the template (*anatomy* and *finding* respectively) based on their ancestries in the ontology (*anatomical entity* and *injury* respectively). The use of composite terms during NER could also give rise to inconsistent annotations, because sub–terms may occur wide apart in text, e.g.

*The < term id = “0000049” > ACL</term > appears chronically < term id = “0000211” > ruptured</term > .*

To address these issues, we simply ignored those branches of the ontology that include composite terms and did not export them from OBO into PathNER’s internal dictionary format. At this point, we addressed two other problems associated with NER, namely ambiguity resolution and recognition of informal names. For example, we noticed that the term *joint effusion* (TRAK:0001410) defined in TRAK as *“Increased fluid in synovial cavity of a joint”* was commonly used in our dataset to refer to its child node *knee effusion* (TRAK:0001411). Safely assuming that in the context of knee MRI reports, *joint effusion* will always refer to *knee effusion*, we ignored the concept identified by TRAK:0001410 and did not export it into PathNER’s dictionary format. Instead, a dictionary entry was created to map *joint effusion* to TRAK:0001411 instead in order for PathNER to recognise its intended meaning within the given context.

Further, we added some new entries to PathNER’s dictionary in order to improve the performance of NER. The reason why such terms were not directly included into the ontology itself is the informal status of such terms (e.g. *tib–fib joint* is an informal synonym of *tibiofemoral joint*), and as such they do not belong to a controlled vocabulary. Given that it is customary for terms to be noun phrases [60], we also limited the use of adjectives and verbs to the leaf nodes of the *finding descriptor* branch as explained earlier. Still, we needed to use these lexical classes as part of NER as we noticed from the training dataset that adjectives and verbs were commonly used to refer to concepts formally described in TRAK. For example, in the following sentence:

*There is a large < term id = “0001089” > lateral meniscal</term><term id = “0001396” > cyst</term > .*

*lateral meniscal* refers to *lateral meniscus* (TRAK:0001089) in which the finding, i.e. *cyst* (TRAK:0001396), is noted, but it would be incorrect to specify it formally as an official synonym of that term within the ontology. Instead, we encoded “unofficial” synonyms separately within PathNER’s dictionary, thus enabling the use of informal synonyms in NER while preserving the strict formality of the ontology. It was in this manner that the verb form *ruptured* was mapped to the term *rupture* (TRAK:0000211) in a previously discussed sentence. In total, the names of 128 concepts were ignored during ontology–to–dictionary conversion and 250 new entries were added to the dictionary.

### Pattern matching

#### Named entity recognition

Following the use of PathNER, a couple of NER–related problems may still persist. For example, consider the following three sentences:

*The < term id = “0000045” > menisci</term > are intact.*

*There are tears in the posterior horns of both < term id = “0000045” > menisci</term > .*

*There is some peripheral signal in both the medial and < term id = “0001089” > lateral meniscus</term > posteriorly.*

with the terms *meniscus* (TRAK:0000045) and *lateral meniscus* (TRAK:0001089) recognised by PathNER. The analysis of their context reveals that all three references should actually be mapped to two concepts: *lateral meniscus* (TRAK:0001089) and *medial meniscus* (TRAK:0001090), both children of *meniscus* (TRAK:0000045). The first two annotations refer to an abstraction of two more specific concepts mentioned, which results in an ambiguous representation of the intended meaning. In the third sentence, correct identification of *medial meniscus* requires the coordinated expression *medial and lateral meniscus* to be interpreted as *medial meniscus and lateral meniscus*. Similarly, dictionary–based NER will fail to recognise enumerated terms. For example, the following sentence mentions three types of *tear* formally described in TRAK:

*This possibly represents a tiny peripheral vertical < term id = “0001390” > longitudinal tear</term > .*

namely, *longitudinal tear* (TRAK:0001390), *vertical tear* (TRAK:0001388) and *peripheral tear* (TRAK:0001389), but only the rightmost one would be recognised by PathNER. Finally, in phrases such as such as *medial meniscectomy*, *patellar tendinitis* and *prepatellar bursitis*, PathNER will succeed in identifying terms referring to findings, i.e. *meniscectomy* (TRAK: 0001511), *tendinitis* (TRAK: 0000229) and *bursitis* (TRAK: 0000225), but it will not recognise implicit references to the anatomical entities affected, i.e. *medial meniscus* (TRAK: 0001090), *patellar tendon* (TRAK: 0000053) and *prepatellar bursa* (TRAK: 0001054).

In KneeTex, these linguistic phenomena are resolved using a set of 109 pattern–matching rules, whose results are used to correct or supplement annotations of named entities generated by PathNER. These rules were implemented in Mixup (My Information eXtraction and Understanding Package), a simple pattern-matching language [61]. For example, the following rules^2^ illustrate the recognition of coordinated references to *medial meniscus*:
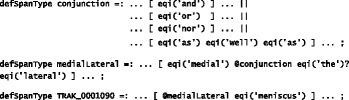


On the training subset of 100 documents, this approach resulted in 430 annotations in addition to 4,439 generated by PathNER, which accounts for approximately 9 % of all named entities recognised.

#### Negation

In addition to supporting NER, pattern matching is used to recognise negation terms: *no*, *not*, *without* and *rather than*. The following examples illustrate their use to negate findings:

*<negation > No</negation > bone marrow < finding > lesion </finding > identified.*

*His patella lies tilted laterally though it has < negation > not </negation > <finding > subluxed </finding > .*

*There is general attenuation of the body of the medial meniscus < negation > without </negation > a discrete < finding > tear</finding > .*

*This represents residual vascularity < negation > rather than</negation > a < finding > tear</finding > .*

Based on our observations on the training data, all negation terms are assumed to occur before the finding they negate. We also defined a single exception to the negation rule. The negation term *no* is ignored when it is used as part of the phrase *no further*, in which case the finding is assumed to be positive, e.g.

*There is a very large < finding > cartilage defect </finding > over the weight bearing surface of the medial femoral condyle. There is no further < finding > cartilage defect</finding > .*

#### Section headings

Although their structure varied across the data set, the given MRI reports generally tended to organise information under the following headings: mri of the left/right knee, indication, history, findings and conclusion. Their lexical and orthographic features were incorporated into a single pattern–matching rule designed to recognize a section heading as a sequence of upper case tokens from a list of fifteen.

### Named entity disambiguation

Once recognised, named entities are imported into a relational database and further scrubbed in order to disambiguate them. Semantic ambiguity may arise naturally from linguistic phenomena such as hyponymy, a relationship between a general term (hypernym) and its more specific instances (hyponyms), and polysemy, where a term may have multiple meanings. Multiple related interpretations may also arise from nested occurrences of named entities.

#### Hyponymy

**Fig. 9:**
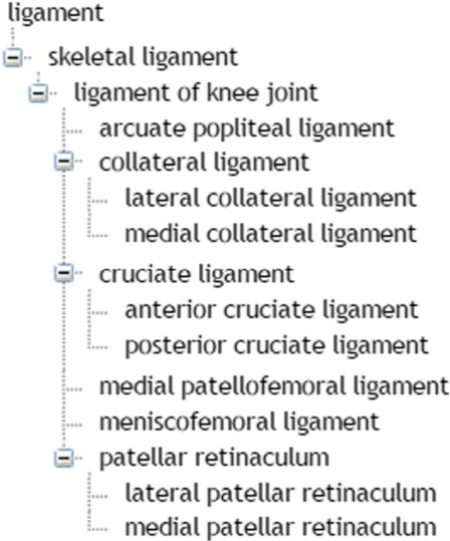
Subclassification of ligaments in the TRAK ontology. The hierarchy is based on *is–a* relationship

When a hypernym such as *ligament* is used, it opens multiple possibilities for its interpretation either as itself in general or any of its hyponyms. Based on the TRAK ontology, a total of 15 concepts may match its intended meaning (see Fig. 9). In a clinical discourse, hypernym and hyponyms are often used as coreferring noun phrases as the means of supporting text coherence and cohesion, e.g.

*There is oedema superficial to the < term id = “0000051” > medial collateral ligament</term > consistent with a sprain but the < term id = “0001027” > ligament</term > is intact.*

In this example, the hypernym *ligament* corefers to its hyponym *medial collateral ligament*, and therefore its interpretation should coincide with that of the hyponym. In other words, the literal interpretation of the hypernym (*ligament*) obtained originally by dictionary lookup should be corrected using the annotation of the coreferring hyponym (*medial collateral ligament*), e.g.

*There is oedema superficial to the < term id = “0000051” > medial collateral ligament</term > consistent with a sprain but the < term id = “0000051” > ligament</term > is intact .*

This type of ambiguity is resolved systematically by identifying coreferential named entities, i.e. those that refer to the same concept. Coreference resolution is applied to named entities recognised as one of the following concepts: *meniscus* (TRAK:0000045), *ligament* (TRAK:0001027) or *tendon* (TRAK:0000046). In such cases, coreference is resolved by looking for previous mentions of their ontological descendants.

#### Polysemy

Sublanguages are restricted to specific semantic domains, which in turn affect the word usage. They generally tend to reduce the degree of polysemy. Nonetheless, the problem may still persist. For example, the word *rupture* in phrases *ligament rupture* and *cyst rupture* would be interpreted differently. In the former case it should be mapped to the following concept in the TRAK ontology:
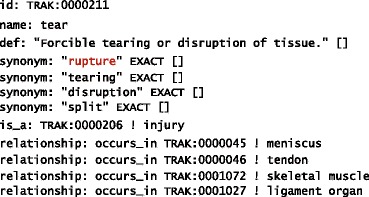


In the latter case, it should be mapped to an alternative interpretation represented by the following concept:
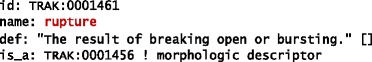


In such cases, co–occurrence information is used to resolve typical ambiguities observed in the training set. For example, when *rupture* co–occurs with a *cyst* (i.e. any descendant of the *cyst* concept), e.g.

*There is oedema in the soft tissues suggesting < term id = “0000211” > rupture</term > of the < term id = “0000222” > popliteal cyst</term > .*

it is used to correct its default interpretation as a *tear*, which represents an *injury*, to an alternative one, which represents a *morphologic descriptor*:

*There is oedema in the soft tissues suggesting < term id = “0001461” > rupture</term > of the < term id = “0000222” > popliteal cyst</term > .*

Thus, we are able to differentiate between the different uses of the term *rupture* in this latter example and that of the following example:

*There is grade 3 < term id = “0000211” > rupture</term > of the MCL.*

#### Nestedness

During dictionary lookup, PathNER will return longest possible matches with similarity scores over a certain threshold. As a result, there will be no overlap between named entities recognised in this manner. However, pattern matching used in the second phase of NER may introduce nested annotations of named entities. For example, in the coordinated expression *medial and lateral meniscus*, PathNER will recognise two terms from the TRAK ontology: *medial* (TRAK:0000031) and *lateral meniscus* (TRAK:0001089). Pattern matching will subsequently recognise a coordinated expression as a reference to *medial meniscus* (TRAK:0001090). The nested occurrence of *lateral meniscus* should be retained as a valid reference to a named entity. However, the nested occurrence of *medial* represents an unsuccessful match to another named entity, *medial meniscus*, and thus should be removed. The choice between retaining and removing nested occurrences of named entities is based on their semantic types. For example, all nested occurrences of terms descending from the concept *quality* (TRAK:0000133) defined as *“a dependent entity that inheres in a bearer by virtue of how the bearer is related to other entities”* are removed. This will remove nested occurrence of *medial* in the previous example, but also references to *radial* (TRAK:0001531) and *vertical* (TRAK:0000077) in the example shown in Table 4.

### Text segmentation

In an effort to separate the contexts in which multiple findings are mentioned within the same sentence of an MRI report, the sentences are divided into segments. Sentence segmentation involves separation of items in lists occurring within certain sections of MRI reports, namely those of history and conclusions. For example,

*historyTwisting injury, tender medial joint line, positive McMurray’s*

would be segmented into three parts relying on comma as a separator. Other sentences are segmented using a set of lexical clues such as *but*, *which*, *consistent with*, etc. For example, the following sentence:

*There is oedema in the soft tissues at the posterolateral corner but the popliteal tendon is intact consistent with a sprain.*

would be separated into three segments:

1. *There is oedema in the soft tissues at the posterolateral corner*
2. *but the popliteal tendon is intact*
3. *consistent with a sprain*

Segmentation greatly simplifies subsequent context analysis. When used in combination with the ontology to infer relationships between named entities, segmentation minimises the need for complex syntactic analysis. In fact, other than analysing prepositional phrases, no other syntactic analysis is performed as part of template filling in KneeTex. Alternatively, syntactic parsing can be used to support text segmentation, but such an approach would be more computationally intensive and not necessarily improving the accuracy. Due to ill formed sentences in clinical narratives, lexical rules may be more robust.

### Template filling

We previously described how the ontology, or more specifically – its vocabulary, is used to support NER as the first step in IE. Template filling as its final step is also driven by the ontology, or more specifically – its structure, i.e. relationships between concepts. This involves accessing information about semantic types by traversing the *is–a* hierarchy in order to identify slot filler candidates. In addition, relationships between the concepts are used to check compatibility between potential slot fillers. For example, if the extracted finding is a *tear*, then the anatomical entity affected must be *soft tissue* such as *ligament* or *tendon*. Similarly, if the affected anatomical entity is *cartilage*, then its qualifier must be related to *bone* or *joint*. We originally considered using OntoCAT for this purpose, as it provides a programming interface to query ontologies shared on BioPortal or user–specified local OBO files [62]. However, this would separate ontology querying from querying data, which are stored in a relational database. In order to enable integrative querying of both data and knowledge, we imported the ontology into the database. This allowed us to implement ontology–driven IE as a series of SQL queries that simultaneously access data and the ontology. The remainder of this section describes the template filling process, where all semantic interpretations mentioned imply the use of such queries.

#### Slot filler candidates

Once the sentences have been segmented, previously recognised named entities are annotated as candidates for specific slots based on their semantic type. Table 7 maps semantic types to the corresponding slots. For example, all named entities identified in the ontology as descendants of *certainty descriptor* (TRAK:0001422) or *visibility descriptor* (TRAK:0001495) are labelled as candidates for filling the *certainty* slot in the template shown in Fig. 1. In addition, co–occurrence of certain concepts and semantic types is used to determine the most appropriate slot filler. For example, when *cartilage* co–occurs with other anatomical concepts, they are labelled as candidates for the *anatomy qualifier* slot rather than the *anatomy* slot as they otherwise would be, e.g.

**Table 7:** Mapping between template slots and semantic types

Slot	Semantic type	TRAK identifier	Example
Finding	Accident	TRAK:0000362	Direct **fall** onto anterior tibia.
	Clinical manifestation	TRAK:0000092	There is some **oedema** superficial to the MCL.
	Modality-related characteristic	TRAK:0001447	The ACL returns **abnormal signal**.
	Morphologic descriptor	TRAK:0001456	There is slight **thickening** of the medial collateral ligament.
	Normality descriptor	TRAK:0001467	The articular cartilage is **unremarkable**.
	Pathological condition	TRAK:0000204	There is a small **Baker’s cyst**.
	Physical examination	TRAK:0000656	Positive **McMurray’s**.
	Physiological condition descriptor	TRAK:0001482	No evidence of articular cartilage **damage**.
	Surgery	TRAK:0000236	Presumably this had been excised during the ACL **reconstruction**.
Finding qualifier	Clinical finding	TRAK:0000091	**Positive** McMurray’s.
	Composition descriptor	TRAK:0001322	Incidental note is made of a **simple** popliteal cyst.
	Distribution pattern	TRAK:0001441	There is **focal** hyaline cartilage fissuring.
	Orientation descriptor	TRAK:0001529	This could represent a **longitudinal** split.
	Quantity descriptor	TRAK:0001468	There are also **several** loose bodies.
	Size descriptor	TRAK:0001485	There is a **small** Baker’s cyst.
	Sport	TRAK:0000323	HISTORY **Squash** injury.
	Stage of healing descriptor	TRAK:0001502	There is a **healing** tear of the medial collateral ligament.
	Status descriptor	TRAK:0001478	Focal area of **severe** chondromalacia in the medial compartment.
	Temporal descriptor	TRAK:0001488	There is **acute** ACL tear.
Certainty	Certainty descriptor	TRAK:0001422	This raises the **possibility** of a previous patella dislocation.
	Visibility descriptor	TRAK:0001495	Normal **appearance** of the articular cartilage.
Anatomy	Anatomical entity	TRAK:0001337	The **menisci** , **collateral ligaments** and the **PCL** are intact.
Anatomy qualifier	Anatomical location descriptor	TRAK:0001561	There is some oedema **superficial** to the MCL.
	General anatomical term	TRAK:0001581	There is a lot of oedema in the ACL **fibres**.
	Meniscus zone	TRAK:0001345	Complex tear of **posterior horn** of the lateral meniscus.

Text in a bold typeset represents instances of a given type

*There is early fissuring and irregularity of the < slot = “anatomy” > hyaline cartilage</slot > of the < slot = “anatomy qualifier” > lateral patellar facet</slot > .*

This is based on an observation that the *finding* will most likely apply to *cartilage* as an object, an observation drawn from the training data.

#### Additional text segmentation

Information about slot filler candidates is used to segment sentences yet again. We previously did not use lexical clues such as *and* or *with* as they do not necessarily indicate another statement about clinical findings, e.g.

*The cruciate and collateral ligaments are <slot=“finding”>intact</slot > .*

However, now that the *finding* slot candidates have been identified, we can combine this information with such lexical clues in order to determine whether to use them to segment a sentence. For example, when two findings are separated by *and* as in:

*There is no <slot=“finding”>oedema</slot> in the lateral femoral condyle and the ACL is <slot=“finding”>intact</slot>.*

then the conjunction is used as a clue to split the sentence into two segments:

1. *There is no <slot=“finding”>oedema</slot> in the lateral femoral condyle*
2. *and the ACL is <slot=“finding”>intact</slot>.*

#### Slot filling

Finally, each segment is analysed in order to fill the template. In the first step, all *findings* are identified within a segment. Following the completion of the two–step segmentation process, most segments will contain at most two findings. The analysis of segments with a single *finding* involves identification of candidates for the following slots: *finding qualifiers, negation, certainty* and *anatomy*, which are all assumed to be linked directly to the given *finding*. Further analysis is required only if there are *anatomy qualifiers* that need to be linked to appropriate *anatomy* slot fillers. Simple analysis of noun phrase structure is used to achieve this. For example, in the following sentence:

*There is marrow oedema at <NP>the <slot=“anatomy qualifier”>medial</slot> aspect of the <slot=“anatomy”>patella</slot></NP> and <NP>the <slot=“anatomy qualifier”>lateral</slot> aspect of the <slot=“anatomy”>lateral femoral condyle</slot></NP>.*

the structure of noun phrases, denoted here by the NP tag, is used to link *anatomy* and *anatomy qualifier* slot fillers.

**Fig. 10:**
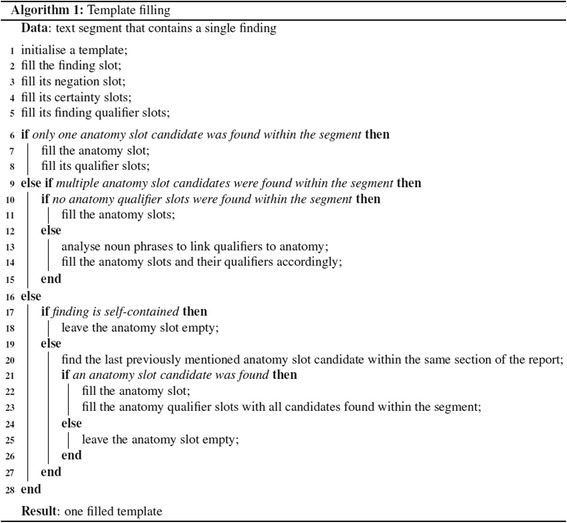
Template filling algorithm. One template is filled for a text segment that contains a single finding. In line 17, a finding is self-contained if it does not require anatomical localisation because it is implicitly stated by the finding itself. For example, Osgood-Schlatter disease is defined as a traction apophysitis of the anterior tibial tubercle. To determine if an extracted finding is self-contained, it is compared against a predefined list

If no *anatomy* filler is found within a segment, an attempt is made to identify potential filler within preceding segments. In the following example:

*There is a vertical longitudinal tear of the peripheral aspect of the posterior third of the <slot=“anatomy”>medial meniscus</slot>. The <slot=“finding”>tear</slot> does not appear significant.*

this approach would result in linking the mention of a *tear* in the second sentence to *medial meniscus* mentioned in the previous sentence. In summary, when a single *finding* is found within a segment, the pseudocode given in Fig. 10 specifies the template filling rules.

When two *findings* are identified within a segment, other slot fillers need to be linked to appropriate *finding*s. Using the end of the first *finding* as a boundary, the remaining slot fillers are divided between the two *findings*. In the following example where a radiologist failed to enter a comma to separate two findings:

*history* *<slot=“anatomy”>Lateral joint line</slot> <slot = “finding” > tenderness</slot > <slot=“anatomy”>meniscal</slot> <slot=“finding”>tear</slot>*

this approach would correctly link *tenderness* to *lateral joint line* and *tear* to *meniscus*. The exception is the use of conjunction *or*, e.g.

**Fig. 11:**
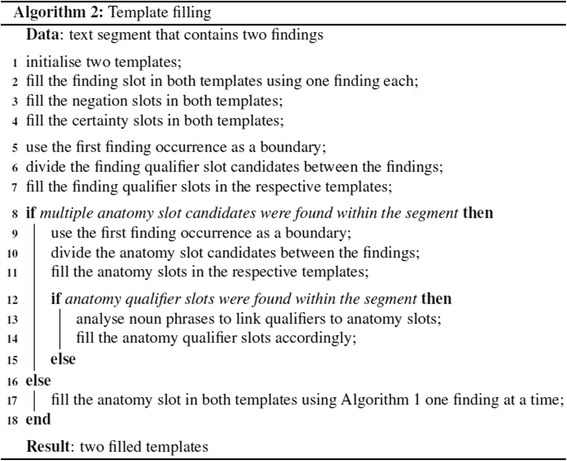
Template filling algorithm. Two templates are filled for a text segment that contains two findings

*There is <slot=“finding”>bone bruising</slot> or <slot=“finding”>subchondral marrow oedema</slot> at the <slot=“anatomy”>inferior patella</a>.*

in which case the slot fillers are shared between the two *findings*. In summary, when two findings are found within a segment, the pseudocode given in Fig. 11 specifies the template filling rules.

## Results

### Gold standard

A test dataset was created as a subset of 100 MRI reports selected randomly from the dataset described previously and removed from consideration prior to system development. Its sole purpose was to test the performance of the system on unseen data. In order to create a gold standard, the test dataset was annotated manually by two independent annotators (see Data).

Fleiss” Kappa coefficient [63] was used to measure the inter–annotator agreement on slot fillers, which were compared at the text span level. It was calculated according to the following formula:

$$ k=\frac{\mathrm{Ao}-\mathrm{A}\mathrm{e}}{1-\mathrm{A}\mathrm{e}} $$

where A_o_ is observed agreement (i.e. the proportion of items on which both annotators agree) and A_e_ is expected chance agreement calculated under the assumption that: (1) both annotators act independently, and (2) random assignment of annotation categories to items, by either coder, is governed by distribution of items across these categories [64]. Fleiss’ Kappa coefficient is measured on a −1 to 1 scale, where 1 corresponds to perfect agreement, 0 corresponds to chance agreement and negative values indicate potential systematic disagreement between the annotators.

**Fig. 12:**
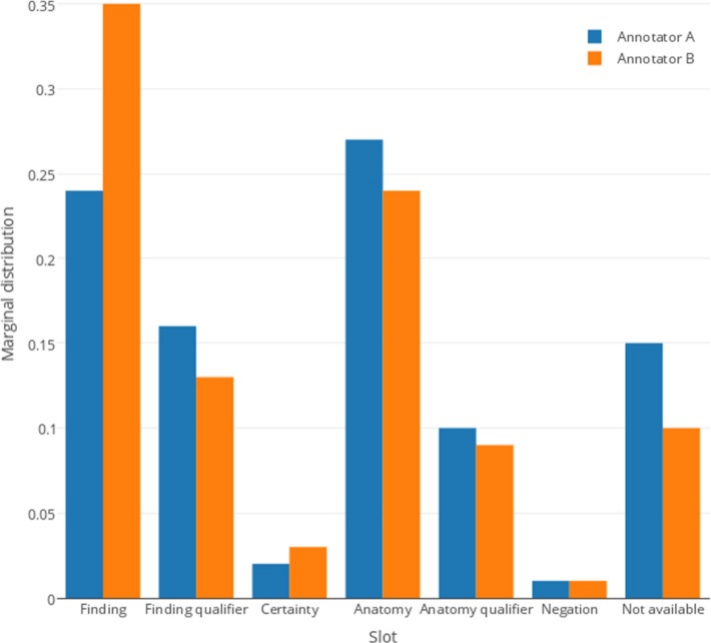
Marginal distribution of annotations across the slots. “Not available” indicates a missing annotation

We calculated the value of Fleiss’ Kappa coefficient using an online tool [65]. The observed and expected agreement were calculated to be *A*_*o*_ = 0.87, and *A*_*e*_ = 0.26 respectively, which resulted in *K* = 0.825 (see Fig. 12 for marginal distribution of annotations across the slots). Following the guidelines for interpreting Fleiss’ Kappa (<0 poor, 0.01–0.20 slight, 0.21–0.40 fair, 0.41–0.60 moderate, 0.61–0.80 substantial, 0.81–1.00 perfect) [66], the given value implies almost perfect agreement.

**Fig. 13:**
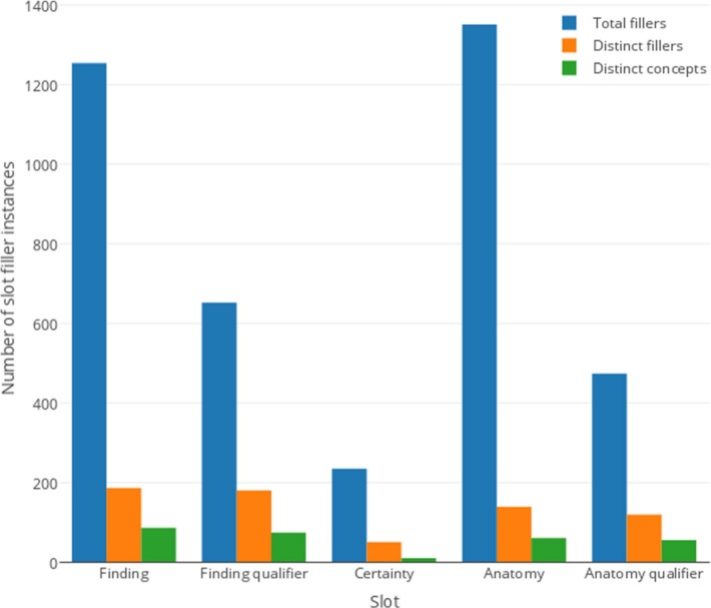
Distribution of annotations in the gold standard. Extracted text and corresponding ontology concepts

A gold standard was created by the third annotator who independently resolved the inter–annotator disagreements, ensured the consistency of annotations and mapped individual annotations of text spans to the corresponding concepts in the TRAK ontology. Gold standard annotations were converted to filled IE templates represented as JSON objects (see Tables 4 and 5 for examples) in order to support their comparison to KneeTex output during evaluation. Figure 13 shows the distribution of slot fillers in the gold standard.

### Evaluation

We evaluated the system at the conceptual level, i.e. we compared manually annotated and automatically extracted mappings to TRAK concepts. In other words, each automatically filled template slot, represented by a TRAK concept identifier, was classified either as a true positive if it matched the slot filler in the gold standard or as a false positive otherwise. Conversely, each slot filler in the gold standard was classified as a false negative if was not extracted by the system. Given the total numbers of true positives (TP), false positives (FP) and false negatives (FN), precision (P) and recall (R) were calculated as the following ratios:

$$ P=\frac{TP}{TP+FP}\kern2em R=\frac{TP}{TP+FN} $$

The performance was evaluated using recall (R) and precision (P), as well as their combination into the F-measure:

$$ F=\frac{2\cdot P\cdot R}{P+R} $$

These values were micro–averaged across each slot (vertical evaluation) as well as the whole entries that take into account the links between the slot fillers (horizontal evaluation). Table 8 provides evaluation results.

**Table 8:** Evaluation results. Performance of the system on the test set

Slot	TP	FP	FN	P	R	F
Finding	1251	5	3	99.60 %	99.76 %	99.68 %
Finding qualifier	636	19	15	97.10 %	97.70 %	97.40 %
Negation	91	1	4	98.91 %	95.79 %	97.33 %
Certainty	232	8	2	96.67 %	99.15 %	97.89 %
Anatomy	1313	30	38	97.77 %	97.19 %	97.48 %
Anatomy qualifier	439	18	34	96.06 %	92.81 %	94.41 %
Overall	3962	81	96	98.00 %	97.63 %	97.81 %

In order to assess the generalizability of the system we conducted a series of stage–wise experiments in which we removed new concepts identified from the training dataset by using strategies 1–3. We specifically focused on concepts outside of the *finding descriptor* class for two reasons. Firstly, this class corresponds to the *RadLex descriptor* branch of the RadLex hierarchy and its dependency on the training data is minimal. Secondly, concepts from this class are used to fill three “leaf” slots (*finding qualifier*, *anatomy qualifier* and *certainty* – see Table 7) that have no further dependencies (see Fig. 1) and as such will have no ripple effect on the template filling unlike *finding* and *anatomy* slots. For example, if *finding* is not identified, it will affect text segmentation as well as linking to other slot fillers. Therefore, the highest impact on evaluation results would be caused by concepts outside the *finding descriptor* branch.

**Fig. 14:**
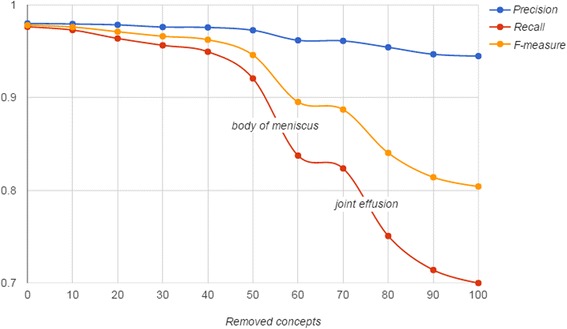
Stage–wise experiments. A total of ten concepts were incrementally removed from the ontology

Having identified just over 100 of such concepts, we randomly selected 100 of them, randomized their order and removed top *k* of these concepts (*k* = 10, 20, … , 100) from the ontology, which was then used to run KneeTex on the gold standard. Figure 14, provides a comparison of evaluation results. As expected, completeness of the ontology directly affected the recall of the system. This was most obvious when frequently referenced concepts such as *body of meniscus* (TRAK:0001346) or *joint effusion* (TRAK:0001411) were removed. However, the frequency and meaning of these concepts imply that they are of general relevance to the domain and not the result of overfitting to the training dataset. On the other side, the removal of less frequently referenced concepts did not have a profound effect on recall. For example, after removing as many as 50 concepts from the ontology, recall was still very high at 92.07 % dropping by 5.57 percent points. Precision proved to be more stable reaching 94.48 % after removing all 100 concepts dropping only by 3.52 percent points.

### Error analysis

#### Dictionary lookup

Some of the errors stem from incorrectly recognised named entities. For example, in segment “*his patella tends to lie tilted laterally,*” string similarity caused PathNER to incorrectly recognise *patella tends* as *patellar tendon*, therefore failing to extract *patella* instead.

#### Coreference

Other errors are the consequence of unresolved coreference. For example, in the text segment “*but there is currently no evidence of a significant meniscal cyst,*” the following slot filler represents correctly extracted text:
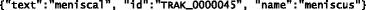


However, if we look at the containing sentence:

*There is a cleavage tear of the lateral meniscus at the junction of the body and posterior horn which extends through the body but there is currently no evidence of a significant meniscal cyst.*

then it becomes clear that the word *meniscal* here co–refers with the previous mention of the *lateral meniscus* and, therefore, should be interpreted as follows:
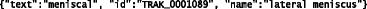


#### Preferred interpretation

Similarly, in sentence “*There is some blunting of the inner edge of the mid portion of the medial meniscus,*” the word *mid* was recognized as a synonym for *middle* (TRAK:0001598). Its classification as an *anatomical location descriptor* (TRAK:0001561) was used to fill the *anatomy qualifier* slot as follows:
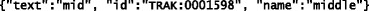


Its literal interpretation as *“an intermediate part or section; an area that is approximately central within some larger region”* happens to be semantically correct. However, in the given context, the following annotation in the gold standard represents preferred interpretation:
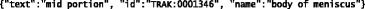


since *body of meniscus* (TRAK:0001346) most specifically represents its middle third.

#### Negation

There were few cases where negation was not recognised. For example, two findings were extracted from the following sentence *“The low signal of the anteromedial bundle seen in a normal ACL is completely absent”*:
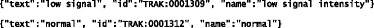


However, the system failed to make use of the clue *absent* found at the end of the sentence to recognise that these findings are actually negative. These errors will be used to inform future improvements of the system.

## Conclusions

In this paper, we described KneeTex, an ontology–driven system for information extraction from narrative reports that describe an MRI scan of the knee. The system exhibited human–level performance on a gold standard. Such performance can be attributed partly to the use of a domain–specific ontology, which serves as a very fine–grained lexico–semantic knowledge base and plays a pivotal role in guiding and constraining text analysis. In this context, the ontology proved to be highly attuned to the given sublanguage. The extent of knowledge engineering involved in the development of domain–specific ontologies with sufficient detail and coverage for text mining applications is known to present a major bottleneck in deep semantic NLP. Therefore, many NLP systems compensate for the lack of suitable semantic resources by resorting to extensive syntactic analysis and heuristic approaches that operate at the level of the textual surface.

We have adopted an alternative approach based on a set of strategies that can be used to systematically expand the coverage of existing ontologies or to develop them from scratch. Three of these strategies are data–driven and as such are more likely to ensure that the ontology effectively supports the intended NLP application. Each data–driven strategy utilises a different approach to extracting the relevant terminology from the data either manually or automatically. The fourth strategy is based on integration of concepts from other relevant knowledge sources. The two main aims of this strategy are: (1) to avoid the overfitting of the ontology to limited data available, and (2) to provide an initial taxonomic structure to incorporate new concepts.

In this study, we illustrated how these strategies were implemented in practice to expand the coverage of the TRAK ontology to make it suitable for a specific NLP application. The evaluation results confirm that KneeTex succeeded in making effective use of the ontology to support IE from knee MRI reports. Previously, we integrated TRAK into web and smartphone applications that provide remote support for knee rehabilitation and for collection of data that can support randomised control trials. Here we have demonstrated how the ontology was repurposed to support an NLP application within a clinical setting. In both cases, formally structured and coded datasets can be easily integrated to support large–scale multi–faceted epidemiologic studies of knee conditions.

## Availability and requirements

**Project name:** KneeTex

**Project home page:**http://www.cs.cf.ac.uk/kneetex

**Operating system(s):** Platform independent

**Programming language:** Java

**Other requirements:** None

**License:** FreeBSD

**Any restrictions to use by non-academics:** None

## Footnotes

1 The values given in brackets refer to standard deviation.

2 Mixup is a pattern language for text spans, i.e. token sequences. Keyword defSpanType defines a span type whose structure is specified to the right of the equal sign, where square brackets [ and ] indicate the start and end of a span respectively, eqi(‘foo’) matches the token foo and … matches any sequence of tokens. Postfix operator ? specifies that the preceding token can be matched either once or not at all. Finally, operator || is used to specify alternative patterns.
